# Supplementary material for: A Systematic Review of Persistent Symptoms and Residual Abnormal Functioning following Acute COVID-19: Ongoing Symptomatic Phase vs. Post-COVID-19 Syndrome
Source: J Clin Med. 2021 Dec 16;10(24):5913. doi: 10.3390/jcm10245913 (PMC8708187; doi:10.3390/jcm10245913)
Supplement: Supplementary file 1 [file jcm-10-05913-s001.zip › jcm-1484275-supplementary.pdf]

Supplementary Data 1. Search Strategy

Embase

'coronavirus disease 2019'/exp

((long OR 'long term' OR 'long lasting' OR Prolonged) NEAR/3 ('2019 ncov' OR ncovor OR covid OR 'sars cov 2')):ti,ab

(('long* haul*' OR longhaul* OR 'long* tail*' OR longtail* OR longduration* OR 'long duration*' OR longlast* OR 'long last*') NEAR/3 ('2019 ncov' OR ncovor OR covid OR 'sars cov 2')):ti,ab

((chronic OR post-infect* OR 'post acute' OR persistent) NEAR/6 ('2019 ncov' OR ncovor OR covid OR 'sars cov 2')):ti,ab

('post covid' OR postcovid OR 'Post acute covid*' OR 'postacute covid*'):ti,ab

#1 OR #2 OR #3 OR #4 OR #5

'hospital discharge'/exp

((long OR 'long term' OR 'long lasting' OR post-acute OR post-covid OR Postdischarge OR Post-discharge OR chronic OR persist* OR linger*) NEAR/3 (effect* OR symptom* OR recover* OR therapy OR sequelae OR complication* OR consequence* OR syndrome* OR outcome*)):ti,ab

((prolong* OR longer OR extended OR lengthy OR lengthen OR increase* OR drawn-out OR proctracted) NEAR/4 recover*):ti,ab

#7 OR #8 OR #9

'fatigue'/de OR 'exhaustion'/de OR 'fatigue assessment scale'/exp OR 'fatigue assessment instrument'/exp OR 'postviral fatigue syndrome'/exp OR 'Fatigue Severity Scale'/exp OR 'Fatigue Impact Scale'/exp

(fatigue* OR tired* OR exhaustion OR exhausted OR lethargy OR sluggish OR 'postviral syndrome' OR 'post viral syndrome' OR 'fatigue severity scale' OR 'myalgic encephalomyelitis'):ti,ab

#11 OR #12

#6 AND #10 AND #13

MEDLINE

COVID-19/

((long OR long term OR long lasting OR Prolonged) adj3 (2019 ncov OR ncovor OR covid OR sars cov 2)).ti,ab.

((long* haul* OR longhaul* OR long* tail* OR longtail* OR longduration* OR long duration* OR longlast* OR long last*) adj3 (2019 ncov OR ncovor OR covid OR sars cov 2)).ti,ab.

((chronic OR post infect* OR post acute OR persistent) adj6 (2019 ncov OR ncovor OR covid OR sars cov 2)).ti,ab.

(post covid OR postcovid OR Post acute covid* OR postacute covid*).ti,ab.

or/1-5

Patient Discharge/

((long OR long term OR long lasting OR post acute OR post covid OR Postdischarge OR Post discharge OR chronic OR persist* OR linger*) adj3 (effect* OR symptom* OR recover* OR therapy OR sequelae OR complication* OR consequence* OR syndrome* OR outcome*)).ti,ab.

((prolong* OR longer OR extended OR lengthy OR lengthen OR increase* OR drawn out OR proctracted) adj4 recover*).ti,ab.

or/7-9

Fatigue/ OR Fatigue Syndrome, Chronic/ OR Muscle Weakness/

(fatigue assessment scale OR fatigue assessment instrument OR Fatigue Severity Scale OR Fatigue Impact Scale).ti,ab.

(fatigue* OR tired* OR exhaustion OR exhausted OR lethargy OR sluggish OR postviral syndrome OR post viral syndrome OR fatigue severity scale OR myalgic encephalomyelitis).ti,ab.

or/11-13

6 AND 10 AND 14

Coronavirus Research Database (ProQuest)

TI ((long OR “long term” OR “long lasting” OR Prolonged) N2 (“2019 ncov” OR ncovor OR covid OR “sars cov 2”)) OR AB ((long OR “long term” OR “long lasting” OR Prolonged) N3 (“2019 ncov” OR ncovor OR covid OR “sars cov 2”))

TI ((“long* haul*” OR longhaul* OR “long* tail*” OR longtail* OR longduration* OR “long duration*” OR longlast* OR “long last*”) N2 (“2019 ncov” OR ncovor OR covid OR “sars cov 2”)) OR AB ((“long* haul*” OR longhaul* OR “long* tail*” OR longtail* OR longduration* OR “long duration*” OR longlast* OR “long last*”) N2 (“2019 ncov” OR ncovor OR covid OR “sars cov 2”))

TI ((chronic OR post-infect* OR “post acute” OR persistent) N5 (“2019 ncov” OR ncovor OR covid OR “sars cov 2”)) OR AB ((chronic OR post-infect* OR “post acute” OR persistent) N5 (“2019 ncov” OR ncovor OR covid OR “sars cov 2”))

TI (“post covid” OR postcovid OR “Post acute covid*” OR “postacute covid*”) OR AB (“post covid” OR postcovid OR “Post acute covid*” OR “postacute covid*”)

1 OR 2 OR 3 OR 4

TI (“fatigue assessment scale” OR “fatigue assessment instrument” OR “postviral fatigue syndrome” OR “Fatigue Severity Scale” OR “Fatigue Impact Scale”) OR AB (“fatigue assessment scale” OR “fatigue assessment instrument” OR “postviral fatigue syndrome” OR “Fatigue Severity Scale” OR “Fatigue Impact Scale”)

TI (fatigue* OR tired* OR exhaustion OR exhausted OR lethargy OR sluggish OR “postviral syndrome” OR “post viral syndrome” OR “fatigue severity scale” OR “myalgic encephalomyelitis”) OR AB (fatigue* OR tired* OR exhaustion OR exhausted OR lethargy OR sluggish OR “postviral syndrome” OR “post viral syndrome” OR “fatigue severity scale” OR “myalgic encephalomyelitis”)

6 OR 7

S5 AND S8

Google Scholar

2019nCoV|"2019 nCoV|CoV|coronavirus"|"2019 novel|new coronavirus|cov"|"wuhan coronavirus|cov|ncov|outbreak"|"wuhan*coronavirus|cov|ncov|outbreak"|"wuhan**coronavirus|cov|ncov|outbreak"|"coronavirus|cov|ncov*wuhan“ fatigue|exhaustion

---

LitCovid

(“long term”[tiab] OR “long lasting”[tiab] OR Prolonged[tiab] OR chronic[tiab] OR post-infect*[tiab] OR “post acute”[tiab]) AND (fatigue*[tiab] OR tired*[tiab] OR exhaustion[tiab] OR exhausted[tiab] OR lethargy[tiab] OR “postviral syndrome”[tiab] OR “post viral syndrome”[tiab] OR “myalgic encephalomyelitis”[tiab])

Supplementary Data 2. Data extraction tool

First Author	Date	Country of Authorship	Primary Topic	Specific Topic(s)	Study Design (re. long COVID)	N	Age (Years)	Gender (% Female)	Eligibility Criteria*	COVID Hospital Status	Time Post-COVID (from recovery/onset)	Weeks from COVID Onset	Fatigue Measurement Tool (FMT)*	Method of FMT Administration
**Arnold**	Apr-21	United Kingdom	Symptomology	1) Symptomatology; 2) Respiratory functioning<br>3) Quality of life; 4) Haemotology	Cross-Sectional	110	**M** = 60<br>**IQR** = 46-73	38%	-	Inpatient	**M** = 83 days<br>**IQR** = 74-88	16 weeks	SF-36 GSQ	Summed rating scale<br>in-person
**Bellan**	Jan-21	Italy	Respiratory	1) Respiratory functioning<br>2) Symptomatology	Cross-Sectional	238	**M** = 61<br>**IQR** = 50-71	40%	-	Inpatient (+ICU)	**All** ~ 4 months	21 weeks	Novel GSQ	Binary scale (Y/N)<br>via phone
**Carfi**	Jul-20	Italy	Symptomology	1) Symptomatology	Short Study	143	**X̄** = 56.5<br>**SD** = 14.6	37%	-	Inpatient (+ICU)	**X̄** = 36.1 days<br>**SD** = 12.9	9 weeks	Novel GSQ	Binary scale (Y/N)<br>in-person
**Carvalho-Schneider**	Oct-20	France	Symptomology	1) Symptomatology	Longitudinal	150	**X̄** = 49.0<br>**SD** = 15.0	56%	**Excl**: ICU or residential care	Mixed (-ICU)	**TI: X̄** = 32.7 days, **SD** = 2.5*<br>**TII: X̄**= 59.7 days, **SD** = 1.7*	5 weeks | 9 weeks	-	-
**Cheng**	Jan-21	United Kingdom	Symptomology	1) Symptomatology<br>2) Respiratory functioning	Cross-Sectional	113	**M** = 73<br>**IQR** = 57-84	44%	-	Inpatient (+ICU)	**R** = 6-12 weeks	13 weeks	Novel GSQ	Binary scale (Y/N)<br>in-person
**Chopra**	Nov-20	USA	Symptomology	1) Symptomatology	Short Study	488	**M** = 62<br>**IQR** = 50-72	48%	-	Inpatient (+ICU)	**All** ~ 60 days	13 weeks	-	-
**Cortés-Telles**	Jun-20	Mexico	Symptomology	1) Symptomatology<br>2) Respiratory functioning	Cross-Sectional	186	**X̄** = 47.0<br>**SD** = 13.0	39%	**Excl**: Critical cases	Mixed	**X̄** = 59.5 days*<br>**SD** = 13.5	9 weeks	Novel GSQ	Binary scale (Y/N)<br>in-person
**Daher**	Oct-20	Germany	Respiratory	1) Symptomatology; 2) Respiratory functioning<br>3) Cognitive/Psych func.; 4) Inflamm. response	Cross-Sectional	33	**X̄** = 64.0<br>**SD** = 3.0	33%	**Incl**: Severe COVID diagnosis<br>**Excl**: ARDS diagnosis & ICU	Inpatient	**M** = 56 days<br>**IQR** = 48-71	12 weeks	Novel GSQ	Binary scale (Y/N)<br>in-person
**D'Cruz**	Jan-21	United Kingdom	Symptomology	1) Symptomatology<br>2) Respiratory functioning	Cross-Sectional	119	**X̄** = 58.7<br>**SD** = 14.4	38%	-	Inpatient (+ICU)	**M** = 61 days<br>**IQR** = 51-67)	13 weeks	Novel GSQ	Binary scale (Y/N)<br>in-person
**De Lorenzo**	Oct-20	Italy	Symptomology	1) Symptomatology	Cross-Sectional	185	**M** = 57<br>**IQR** = 48-67	34%	-	Mixed	**M** = 23 days<br>**IQR** = 20-29	7 weeks	-	-
**Froidure**	Apr-21	Belgium	Respiratory	1) Respiratory functioning<br>2) Symptomatology	Cross-Sectional	134	**M** = 60<br>**IQR** = 53-68	41%	**Excl**: Follow-ups 60<x<120 days	Inpatient (+ICU)	**M** = 95 days<br>**IQR** = 86-107	18 weeks	Novel GSQ	Binary scale (Y/N)<br>in-person
**Garrigues**	Aug-20	France	Symptomology	1) Symptomatology	Short Study	120	**X̄** = 63.2<br>**SD** = 15.7	37%	-	**Multiple Cohorts**:<br>Inpatient | ICU	**X̄** = 110.9 days*<br>**SD** = 11.1	16 weeks	Novel GSQ	Binary scale (Y/N)<br>via phone
**Halpin**	Feb-21	United Kingdom	Symptomology	1) Symptomatology; 2) Rehabilitation needs<br>3) Psych. wellbeing; 4) Quality of life; 5) Other	Cross-Sectional	100	**M** = 70.5 / 58.5<br>**R** = 20-93 / 34-84	46%	**Incl**: Discharged<br>**Excl**: Comorbid neuro. condition	**Multiple Cohorts**:<br>Inpatient | ICU	**X̄** = 48.0 days<br>R = 29-71	11 weeks	Novel GSQ	Likert scale<br>via phone
**Huang (I)**	Jan-21	China	Symptomology	1) Symptomatology; 2) Respiratory functioning<br>3) Quality of life; 4) Bloods	Cross-Sectional	1,733	**M** = 57<br>**IQR** = 47-65	48%	**Excl**: Comorbid psych. Condition; Re-admission; Immobility issues	Inpatient (+ICU)	**M** = 153 days<br>**IQR** = 146-160	26 weeks	Novel GSQ	Binary scale (Y/N)<br>in-person
**Huang (II)**	Jun-20	China	Respiratory	1) Respiratory functioning	Cross-Sectional	57	**X̄** = 46.7<br>**SD** = 13.8	54%	**Excl**: Previous pulmonary resection, neurological disease, or mental illness	Inpatient	**All** = 30 days	8 weeks	-	-
**Iqbal**	Feb-21	Pakistan	Symptomology	1) Symptomatology	Cross-Sectional	158	**X̄** = 32.1<br>**SD** = 12.4	55%	**Excl**: Psychiatric illness	Mixed	**X̄** = 38.1 days<br>**SD** = 20.0	7 weeks	Novel GSQ	Binary scale (Y/N)<br>via phone
**Jacobs**	Dec-20	USA	Symptomology	1) Symptomatology<br>2) Quality of life	Longitudinal	183	**M** = 57<br>**IQR** = 48-68	38%	**Incl**: Hospital for 3+ days<br>**Excl**: Dementia/Delirium	Inpatient	**TI** = 0 days | **T2** = 14 days | **TIII** = 21 days | **TIV** = 35 days (IQR=30-40)	4 weeks | 6 weeks | 7 weeks | 9 weeks	PROMIS GSQ	Likert scale<br>via phone/email
**Lerum**	Apr-21	Norway	Respiratory	1) Respiratory functioning	Cross-Sectional	103	**M** = 59<br>**IQR** = 49-72	48%	-	**Multiple Cohorts**:<br>Inpatient | ICU	**M** = 83 days*<br>**IQR** = 73-90	12 weeks	-	-
**Liang**	Oct-20	China	Symptomology	1) Symptomatology<br>2) Respiratory functioning	Cross-Sectional	76	**M** = 41.3<br>**IQR** = 13.8	72%	**Excl**. Comorbid neuro. condition; Underwent pulmonary resection	Inpatient (+ICU)	**Ti** = 1 month, **TII** = 2 months, **TIII** = 3 months; **SD** = 1	5 weeks | 13 weeks | 17 weeks	Novel GSQ	Binary scale (Y/N)<br>in-person
**Loerinc**	Mar-21	USA	Other	1) Transition of COVID care at discharge<br>2) Symptomatology	Cross-Sectional	310	**M** = 58<br>**Range** = 23-99	51%	-	Inpatient (+ICU)	**All** = Discharge	4 weeks	Novel GSQ	Obtained from physician discharge notes
**Mandal**	Sep-20	United Kingdom	Symptomology	1) Long COVID<br>2) Respiratory functioning	Cross-Sectional	384	**X̄** = 59.9<br>**SD** = 16.1	38%	-	Inpatient (+ICU)	**M** = 54 days<br>**IQR** = 47=59	12 weeks	Novel GSQ	Likert scale<br>via phone / in-person
**Miyazato**	Oct-20	Japan	Symptomology	1) Symptomatology	Longitudinal	63	**X̄** = 48.1<br>**SD** = 18.5	33%	-	Inpatient	**X̄** = 108.0 days, **SD** = 23.0<br>(**TI** ~ 60 days, **TII** ~ 120 days)*	9 weeks | 17 weeks	Novel GSQ	Binary scale (Y/N)<br>via phone
**Mo**	Jun-20	China	Respiratory	1) Respiratory functioning	Short Study	110	**X̄** = 49.1<br>**SD** = 4.0	50%	**Excl**: Critical cases	Inpatient	**All** = Discharge	4 weeks	-	-
**Moreno-Pérez**	Mar-21	Spain	Symptomology	1) Symptomatology<br>2) Respiratory functioning	Cross-Sectional	277	**M** = 62<br>**IQR** = 53-72	47%	-	Mixed	**M** = 77 days*<br>**IQR** = 71-85	11 weeks	Novel GSQ	Binary scale (Y/N)<br>in-person
**Osikomaiya**	Mar-21	Nigeria	Symptomology	1) Symptomatology	Cross-Sectional	274	**X̄** = 41.8<br>**SD** = 11.8	34%	-	Outpatient	**M** = 15 days<br>**IQR** = 14-17	6 weeks	Novel GSQ	Binary scale (Y/N)<br>in-person
**Prieto**	Mar-21	Argentina	Symptomology	1) Symptomatology	Cross-Sectional	85	**X̄** = 43.0<br>**SD** = 13.0	45%	-	Mixed	**M** = 53 days*<br>**IQR** = 31-65	8 weeks	Novel GSQ	Binary scale (Y/N)<br>in-person
**Raman**	Nov-20	United Kingdom	Symptomology	1) Symptomatology; 2) Neurological functioning<br>3) Respiratory func.; 4) Psych. Wellbeing; Other	Cross-Sectional	58	**X̄** = 55.4<br>**SD** = 13.2	41%	**Incl**: Moderate-to-severe COVID	Inpatient (+ICU)	**M** = 2.3 months*<br>**IQR** = 2.06-2.53	10 weeks	Fatigue Severity Scale	Likert scale<br>in-person
**Rosales-Castillo**	Jan-21	Spain	Symptomology	1) Symptomatology<br>2) Psychological wellbeing	Cross-Sectional	118	**X̄** = 60.2<br>**SD** = 15.1	44%	-	Inpatient	**X̄** = 50.8 days<br>**SD** = 6.0	11 weeks	Novel GSQ	Binary scale (Y/N)<br>in-person
**Shah**	Mar-21	Canada	Respiratory	1) Respiratory functioning<br>2) Symptomatology	Cross-Sectional	60	**M** = 67<br>**IQR** = 54-74	32%	-	Inpatient	**X̄** = 11.7 weeks*<br>**SD** = n/a	12 weeks	-	-
**Simani**	Feb-21	Iran	Symptomology	1) Symptomatology<br>2) Psychological wellbeing	Cross-Sectional	120	**X̄** = 54.6<br>**SD** = 16.9	33%	**Excl**: Previous life-threatening infection	Inpatient (+ICU)	**All** ~ 6 months	30 weeks	Fukuda questionnaire	Likert scale<br>via phone
**Sykes**	Apr-21	United Kingdom	Symptomology	1) Symptomatology; 2) Haemotology<br>3) Respiratory functioning	Cross-Sectional	134	**M** = 58<br>**R** = 25-89	34%	**Excl**: Mild symptoms or frail	**Multiple Cohorts**:<br>Inpatient | ICU	M = 113 days, R = 46-167<br>(TI~61, TII~88, TIII~113, TIV~147)	13 weeks | 17 weeks| 20 weeks | 25 weeks	Novel GSQ	Binary scale (Y/N)<br>in-person
**Taboada**	Dec-20	Spain	Quality of Life	1) Quality of life<br>2) Symptomatology	Cross-Sectional	91	**X̄** = 65.5<br>**SD** = 10.4	35%	-	ICU	**All** ~ 6 months	30 weeks	-	-
**Townsend (I)**	Nov-20	Ireland	Symptomology	1) Symptomatology<br>2) Haemotology	Cross-Sectional	128	**X̄** = 49.5<br>**SD** = 15.0	54%	**Incl**: 6+ weeks post-COVID	Mixed	**M** = 72 days<br>**IQR** = 62-87	14 weeks	Chalder Fatigue Scale<br>(incl. Psych/Phys sub-scales)	Likert scale<br>in-person
**Venturelli**	Jan-21	Italy	Respiratory	1) Symptomatology	Cross-Sectional	767	**X̄** = 63.0<br>**SD** = 13.6	33%	-	Inpatient (+ICU)	**M** = 105 days*<br>**IQR** = 84-127	15 weeks	Novel GSQ	Binary scale (Y/N)<br>in-person
**Walle-Hansen**	Mar-21	Norway	Quality of Life	1) Quality of life	Cross-Sectional	106	**X̄** = 74.3<br>**R** = 60-96	43%	-	Inpatient (+ICU)	**M** = 186<br>**IQR** = n/a	31 weeks	-	-
**Wang**	May-20	China	Symptomology	1) Symptomatology; 2) Respiratory functioning<br>3) Haemotology	Longitudinal	131	**M** = 49<br>**IQR** = 36-62	55%	**Incl**: Objective improvements (incl. fever, chest CT, respiratory symptoms)	Inpatient	**TI** = 0 days, **TII** = 7-14 days<br>**TIII** = 21-28 days	4 weeks | 6 weeks | 8 weeks	Novel GSQ	Binary scale (Y/N)<br>in-person
**Wong**	Nov-20	Canada	Symptomology	1) Symptomatology	Short Study	78	**X̄** = 62.0<br>**SD** = 16.0	36%	-	Inpatient	**M** = 13 weeks*<br>**IQR** = 11-14	13 weeks	-	-
**Xiong**	Sep-20	China	Symptomology	1) Symptomatology<br>2) Psychological wellbeing	Cross-Sectional	538	**M** = 52<br>**IQR** = 41-62	55%	**Incl**: 3+ months post-COVID	Inpatient	**M** = 97 days<br>**IQR** = 95-102	18 weeks	Novel GSQ	Binary scale (Y/N)<br>via phone
**Yu**	Mar-20	China	Respiratory	1) Respiratory functioning	Cross-Sectional	32	**M** = 44.4<br>**IQR** = N/A	31%	**Incl**: 3 chest CT scans (2-hospital; 1-post)	Inpatient (+ICU)	**M** = 9 days<br>**IQR** = N/A	5 weeks	Novel GSQ	Binary scale (Y/N)<br>in-person
									**excl. COVID+ test result, age & hospital status*		** = from symptom onset or hospitalisation*<br><br>*[blank] = from hospital discharge or COVID recovery*	**"From COVID onset" = +28 days until "recovery"*	*Novel GSQ = Novel general symptom questionnaire*	
